# Organic acids from root exudates of banana help root colonization of PGPR strain *Bacillus amyloliquefaciens* NJN-6

**DOI:** 10.1038/srep13438

**Published:** 2015-08-24

**Authors:** Jun Yuan, Nan Zhang, Qiwei Huang, Waseem Raza, Rong Li, Jorge M. Vivanco, Qirong Shen

**Affiliations:** 1 Key Laboratory of Plant Nutrition and Fertilization in Lower-Middle Reaches of the Yangtze River, Ministry of Agriculture; Jiangsu Collaborative Innovation Center for Solid Organic Waste Resource Utilization; Nanjing Agricultural University, Nanjing, 210095, China; 2 Department of Horticulture and Landscape Architecture and Center for Rhizosphere Biology, Colorado State University, Fort Collins, Colorado 80523

## Abstract

The successful colonization of plant growth promoting rhizobacteria (PGPR) in the rhizosphere is an initial and compulsory step in the protection of plants from soil-borne pathogens. Therefore, it is necessary to evaluate the role of root exudates in the colonization of PGPR. Banana root exudates were analyzed by high pressure liquid chromatography (HPLC) which revealed exudates contained several organic acids (OAs) including oxalic, malic and fumaric acid. The chemotactic response and biofilm formation of *Bacillus amyloliquefaciens* NJN-6 were investigated in response to OA’s found in banana root exudates. Furthermore, the transcriptional levels of genes involved in biofilm formation, *yqxM* and *epsD*, were evaluated in response to OAs via quantitative reverse transcriptase polymerase chain reaction (qRT-PCR). Results suggested that root exudates containing the OAs both induced the chemotaxis and biofilm formation in NJN-6. In fact, the strongest chemotactic and biofilm response was found when 50 μM of OAs were applied. More specifically, malic acid showed the greatest chemotactic response whereas fumaric acid significantly induced biofilm formation by a 20.7–27.3% increase and therefore biofilm formation genes expression. The results showed banana root exudates, in particular the OAs released, play a crucial role in attracting and initiating PGPR colonization on the host roots.

PGPR is a collective term used to describe a group of beneficial bacteria capable of colonizing the rhizosphere leading to the stimulation of plant growth and/or the protection of plants from soil-borne phytopathogens^1^. Plant growth promoting mechanisms include the production of antimicrobial compounds such as lipopeptides and polyketides which are bioactive against phytopathogens^2^,^3^; the secretion of phytohormones such as indole acetic acid and gibberellic acid, which stimulate plant growth^4^,^5^, induction systemic resistance against pathogens^6^ and the production of bioactive volatile organic compounds^7^,^8^. However, in order for plants to acquire the beneficial effects from PGPR, as previously mentioned, it is crucial for PGPR to colonize the plant root system^9^,^10^. Therefore, it is important to understand the deeper processes involved in the colonization of roots by PGPR, such as the detailed physiology and molecular mechanisms.

Colonization is one of the important elements of plant-PGPR interactions before PGPR may exert biocontrol effects against diseases and promote plant growth. However, prior to root colonization, chemotaxis of PGPR towards the roots system is mandatory^11^. Many root-colonizing *Bacillus* strains have been used as biocontrol agents and/or to induce plant growth^12^,^13^,^14^. In addition, once the PGPR reach the root system, biofilm formation is a prerequisite for successful root colonization by microorganisms^12^. Biofilms are the conglomeration of microbial cells attached to a surface and enclosed by extracellular polymeric materials^15^,^16^. Numerous components of the bacterial cell surface such as cell wall polysaccharides, extracellular proteins and exopolysaccharide can aid in the root attachment process^17^. After PGPR colonize the root surfaces, they proliferate and reproduce by receiving key signaling compounds and nutrients from the root exudates which subsequently leads to biofilm formation on the root system^18^. Biofilm formation on the roots is indicative of successful plant-PGPR colonization.

Root exudates are perceived as the first line of communication between roots and PGPR in the rhizosphere^19^,^20^ and are composed of amino acids, organic acids (OAs), phenolics, sugars, and proteins^20^,^21^. Root exudates not only provide PGPR with a source of nutrition, but also serve as signals that can attract and/or repel microorganisms^18^. Root exudates at different stages of plant growth are unique and these distinct exudates could specifically modulate the rhizosphere microbiome^22^,^23^. It has been reported that low molecular weight OAs such as malic, citric and fumaric acid released by roots play initial roles in the recruitment of PGPR to the roots serving as carbon substrates and signaling molecules^21^,^24^,^25^,^26^. Organic acids, especially those involved in the tricarboxylic acid cycle have been shown to play functional roles as carbon and molecular signaling sources^27^.

*Bacillus amyloliquefaciens* NJN-6, isolated from the rhizosphere of banana plants has shown to protect its host from *Fusarium oxysporum* f. sp. *cubense* and promotes plant growth^4^. In addition, the NJN-6 strain has shown to colonize the roots and produce several active compounds^4^. However, the role of banana root exudates and the interaction of root colonization with the NJN-6 strain were yet to be determined and is therefore the focus of this present study. In order to elucidate the detailed mechanisms involved, the OAs released by the banana exudates were analyzed with the aid of high pressure liquid chromatography (HPLC). As chemotaxis and biofilm formation are known to be crucial for rhizosphere colonization of PGPRs, the effects of the OAs produced by the root exudates on chemotactic responses and biofilm formation of strain NJN-6 were evaluated. Results indicated that the malic acid and fumaric acid in root exudates could attract NJN-6 and promoted its biofilm formation by activating relevant genes.

## Results

### NJN-6’s response to banana root exudates as measured by chemotaxis and biofilm formation

**Figure 1:**
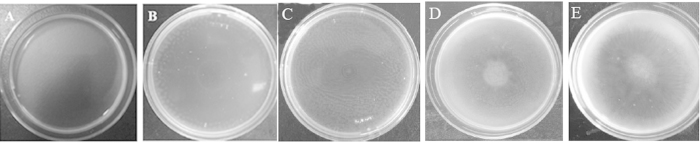
Chemotactic response of *Bacillus amyloliquefaciens* NJN-6 towards banana root exudates and OAs. (**A**) chemotaxis buffer; (**B**) concentrated root exudates of banana; (**C**) oxalic acid; (**D**) malic acid; (**E**) fumaric acid.

**Figure 2:**
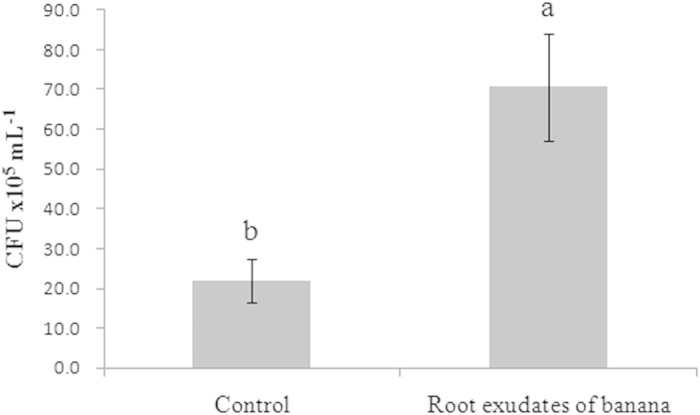
Chemotactic response of NJN-6 towards concentrated root exudates of banana. Bars indicate the standard errors of the means from three replicates. Columns with different letters are statistically different according to the Duncan test (*p* < 0.05).

**Figure 3:**
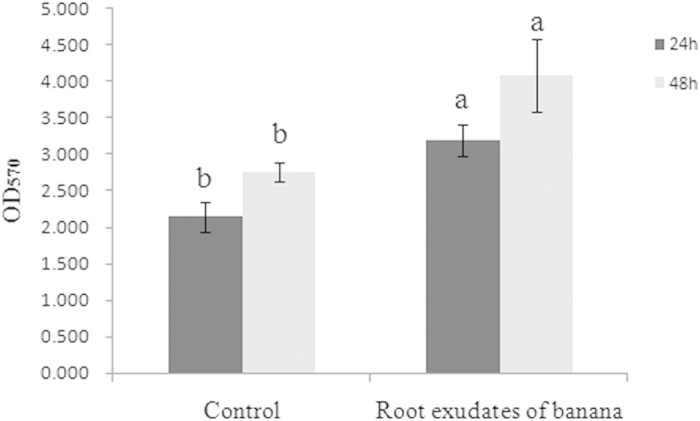
Biofilm formation of NJN-6 in 1/2 MSgg induced by concentrated root exudates of banana. Bars indicate the standard errors of the means from three replicates. Columns with different letters are statistically different according to the Duncan test (*p* < 0.05) in incubation time 24 h or 48 h.

In a qualitative assay, the concentrated root exudates attracted the cells of strain NJN-6 (Fig. 1A and Fig. 1B) and this attraction was three times more than the control (no root exudates) (Fig. 2). The OAs (oxalic acid, malic acid and fumaric acid) also induced chemotaxis on the NJN-6 strain in the drop assay (Fig. 1C–E). The banana root exudates also stimulated biofilm formation of NJN-6 strain in ½ MSgg medium by 28.1% as compared to the control (Fig. 3).

### OAs analysis of root exudates by HPLC

**Figure 4:**
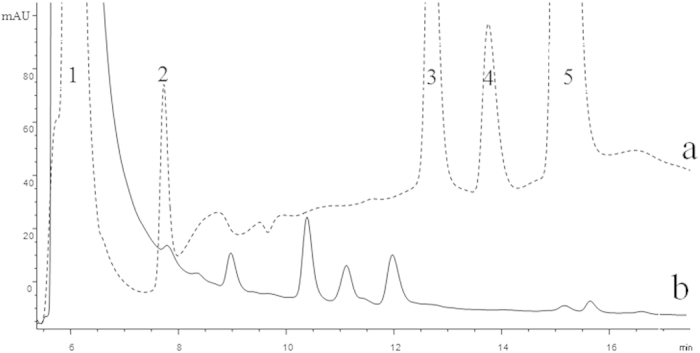
The chromatogram of OAs detected by HPLC. (**a**) The standard compounds: 1, oxalic acid; 2, malic acid; 3, citric acid; 4, succinic acid; 5, fumaric acid. (**b**) Root exudates of banana.

In order to investigate the components of the root exudate which may show the attractant effects on the NJN-6 strain, the OAs were detected by HPLC. Five OAs (oxalic acid, malic acid, citric acid, succinic acid, and fumaric acid) were used as standard samples (Fig. 4a). Oxalic acid, fumaric acid, and malic acid were identified by comparing the retention times with standard samples (Fig. 4b). All these corresponding peaks were analysed by LC-MS for confirmation. The concentration of oxalic acid in the root exudates was significant higher than the other two OAs.

### Quantitative measurement of chemotactic response and biofilm formation by NJN-6

**Figure 5:**
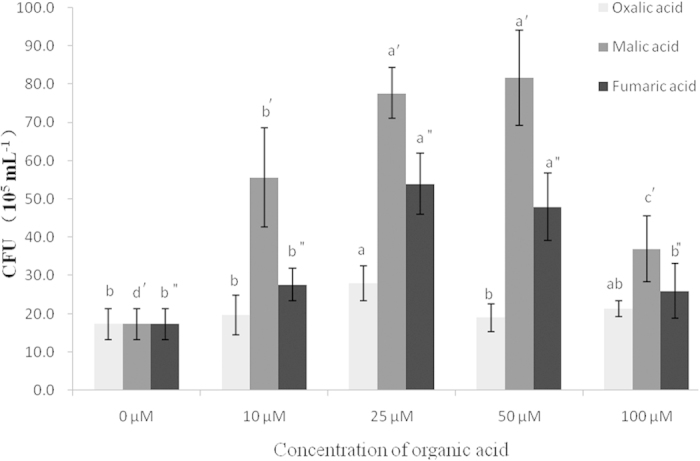
Chemotactic response of NJN-6 towards various detected OAs with different concentrations evaluated by capillary assay. Bars indicate the standard errors of the means from three replicates. Columns with different letters are statistically different according to the Duncan test (*p* < 0.05) among each com*p*ound treatment.

The capillary assay was set up to measure quantitatively the chemotactic response of the NJN-6 strain towards the three OAs identified in the banana root exudates. Malic acid showed the most significant attraction for NJN-6 strain at a 25 or 50 μM concentration (Fig. 5). Fumaric acid at the concentrations of 25 and 50 μM also attracted the NJN-6 strain compared to the control. However, the chemotactic response of NJN-6 strain towards oxalic acid was only visible at a concentration of 25 μM. Only fumaric acid at concentrations of 25 and 50 μM significantly stimulated biofilm formation by the NJN-6 strain which showed an increase of 27.3 and 20.7% compared to the control, respectively (Table 1). The effects of the other two OAs (oxalic acid and malic acid) was not different from the control.

### Transcription analysis of biofilm formation genes

**Figure 6:**
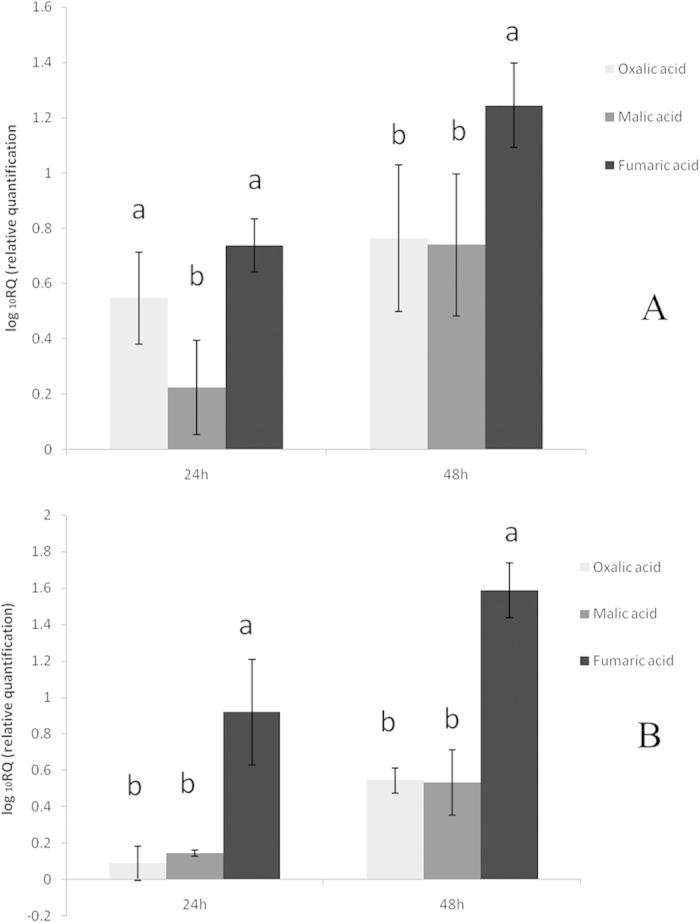
Transcription of biofilm formation genes: *yqxM* and *epsD* in *B. amyloliquefaciens* NJN-6 evaluated by qRT-PCR. The experiments were carried out with cell aggregates obtained from microtiter plates after the cells were grown in MSgg medium for 12 h. The *B. amyloliquefaciens* NJN-6 *recA* gene was used as an internal reference gene. (**A**) *epsD* gene; (**B**) *yqxM* gene. Bars indicate the standard errors of the means from four replicates. Columns with different letters are statistically different according to the Duncan test (*p* < 0.05).

The genes *yqxM* and *epsD* were involved in the production of TasA and exopolysaccharide (EPS), which are essential for biofilm formation. We therefore evaluated the transcriptional levels of these two genes related to biofilm formation, *epsD* and *yqxM,* in *B. amyloliquefaciens* NJN-6. Results indicated that fumaric acid showed the highest level of *epsD* transcription compared to the other OAs at 48 h (Fig. 6A). Fumaric acid also behaved in a similar way with regard to *yqxM* at both time points, suggesting it promoted the biofilm formation of NJN-6 by activation of the two relevant genes (Fig. 6B).

## Discussion

Root exudates play an important role in regulating the bioactivities which occur in the rhizosphere^28^. We previously reported that the *B. amyloliquefaciens* NJN-6 strain showed an enhanced colonization of the banana root surface when plants were grown in vermiculite under sterile conditions^4^. Results from this current study (Figs 1, 2, and 3) showed that the chemotactic response and biofilm formation of the NJN-6 strain was induced in the presence of banana root exudates. Other reports have highlighted the role of root exudates in PGPR root colonization^15^,^25^,^29^.

Chemotaxis is a vital motility property that allows bacteria tomove towards the surface of roots; chemotaxis towards root exudates has been suggested to be the first step of bacterial colonization^11^. In our studies, malic acid induced strong chemotaxis on the NJN-6 strain. *Arabidopsis* roots were reported to recruit beneficial microbes from the rhizosphere by secreting attractant malic acid^25^. Similar results were also found for other PGPR such as *Pseudomonas fluorescens*^21^ and *Paenibacillus polymyxa*^26^. Furthermore, among all the detected OAs in the tomato root exudates citric acid and malic acid were reported as the main chemo-attractants for *P. fluorescens* WCS365 cells^21^.

Chen *et al.*^30^ found that L-malic acid could induce biofilm formation of *Bacillus subtilis* NCIB3610 strain via a KinD-Spo0A dependent pathway, and Rudrappa *et al.*^25^ also reported this phenomena by the induced expression of *yqxM*. We identified that the expression levels of *yqxM* and *epsD* were increased in the presence of malic acid (Fig. 6), but no significant increase inbiofilm biomass was detected (Table 1). In the biofilm formation process, *epsD* and *yqxM* are two pivotal biosynthesis genes for synthesis of exopolysaccharide (EPS) and TasA protein, respectively^31^. In our research, fumaric acid was involved in both attraction and induction of biofilm formation, and could stimulate the expression of biofilm formation related genes.

In our studies, oxalic acid did not show high chemotaxis or biofilm formation, though its concentration in the root exudates was significant higher than the other two OAs. However, oxalic acid has been reported to only affect the swarming of some bacteria^26^ but there are no reports in regards to chemotaxis and biofilm formation.

Chemotaxis to rhizosphere and biofilm formation on plant roots are recognized as two continuous steps for PGPRs to successfully colonize on plant roots and exert the beneficial roles^32^, while most of the previous studies regarding root exudates-governed plant-microbes interactions focused on one aspect of this process^11^,^21^,^30^. However, here we provide a possible mechanism which may explain the increase in colonization of PGPR strain NJN-6 on banana roots, could be attributed to both induction of chemotaxis and biofilm formation by the OAs in the root exudates. Among the three low-molecular weight OAs detected in banana root exudates, malic acid showed signifciant attractive effect on *B. amyloliquefaciens* NJN-6, while fumaric acid induced a significant change in biofilm formationof NJN-6 strain, probably through acitvation of biofilm formation related genes. Combining the good chemotactic response and biofilm formation of the NJN-6 strain towards banana root exudates, it was concluded that OAs play an important role in plant-PGPRs interaction. Further studies could focused on identification of chemotactic receptors for sensing the specifc OAs signal, and exploring the that in what detail fumaric acid activate the biofilm formation relevant genes.

## Materials and Methods

### Microorganism, culture medium and growth conditions

*Bacillus amyloliquefaciens* strain NJN-6 (CGMCC accession number 3183, China General Microbiology Culture Collection Center) that isolated from banana rhizosphere and revealing outstanding suppression effects against *Fusarium oxysporum* f. sp. *cubense*^33^, was incubated at 37 °C, 170 rpm in Luria-Bertani (LB) medium. Half strength MSgg medium [2.5 mM potassium phosphate (pH 7), 50 mM MOPS (pH 7), 25 μM MnCl_2_, 350 μM CaCl_2_, 1 mM MgCl_2_, 50 μM FeCl_3_, 0.5 μM ZnCl_2_, 0.25% glutamate, 0.25% glycerol, 1 μM thiamine, 25 μg·mL^−1^ phenylalanine, 25 μg·mL^−1^ tryptophan, and 25 μg·mL^−1^ threonine]^34^ was used as primary medium for the biofilm formation assay.

### Plant growth conditions and root exudate collection

Banana plant (Musa AAA Cavendish cv. Brazil) seedlings originated from tissue culture were provided by Wanzhong Industrial Limited Company (Hainan province, China). Plants were cultured in sterilized vermiculite at 28 °C with a 16-h light/8-h dark photoperiod and irrigated with sterile liquid half strength Hoagland medium (sucrose-free). After 15 days of growth, plants with 3–4 leaves were uprooted from the substrate and the roots were gently washed with sterilized water to remove the adhered vermiculite.

*In vitro* grown banana plants were transplanted into 100 ml flasks containing 50 ml sterilized double-distilled water for root exudate collection for two days under as described above. Root exudate solution (1500 mL) was collected from 30 seedlings, and then filtered through 0.45 μm a polytetrafluoroethylene (PTFE) membrane. The filtered exudates were lyophilized and resolved in 30 mL water (50 times concentrated) and stored at −80 °C for further experimentation.

### Analysis of OAs in root exudates

To analyze low molecular weight organic acids, an HPLC XDB-C_18_ (4.6 × 250 mm, Agilent, Santa Clara, CA, USA) was used. The mobile phase consisted of water with 5 mM H_2_SO_4_ (A) and 100% methanol (B) with a gradient elution of 0.4 mL min^−1^. The composition of the gradient was as follows: from 0 min to 10 min, the mobile phase was 95% A with 5% B, then the mobile phase changed to 90% A with 10% B for the next 50 min, and the whole process lasted for 60 min^26^. An ultraviolet (UV) detector was used to detect peaks at 210 nm. Five organic acids that reported to be the important components in plant root exudates^26^, including malic acid, oxalic acid, succinic acid, citric acid, and fumaric acid, were selected as the standards. These standard organic acids (purchased from Sigma, USA) and root exudates were injected (10 μL) into the chromatographic system sequentially and run under the same conditions. Three replications were carried out for each sample. OAs in the root exudate was identified by comparison with the retention time of standard samples.

In order to identify the OAs, the liquid chromatography-mass spectrometry (1200 series, Agilent, Santa Clara, CA, and ESI–MS, 6410 Triple Quad LC/MS, Agilent, Santa Clara, CA) was also used to identify and confirm the collected peaks from both samples. The column used was a C_18_ column (50 × 2.1 mm, 1.8 μm) adjusted to a flow rate of 0.4 mL min^−1^ and the mobile phase consisted of H_2_O (A) and 100% methanol (B) with the HPLC elution gradient as reported.

### Chemotaxis assay

The “drop” assay described by de Weert *et al.*^21^, with slight modifications, was performed for quantification of chemotaxis. Briefly, the strain NJN-6 was grown in LB medium at 37 °C and 170 rpm until an OD_600_ (Spectra Max M5 analysis system, Molecular Devices Corporation, CA, USA) of 0.8 was reached. Cells grown in 50 ml media were collected by centrifugation and then resuspended in 12 mLof chemotaxis buffer (100 mM potassium phosphate [pH 7.0] with 20 μM EDTA). A 4 mL aliquot of 1% hydroxypropylmethylcellulose solution was added to the cell suspension. A 60-mm-diameter Petri dish was used to contain the cell suspension. Concentrated root exudate or individual OAs (10 μL for a drop, 0.1 M) were added to the center of each Petri dish. A ring of turbidity near the center of each Petri dish would appear after an incubated of 10–15 min at room temperature (RT), if the chemotactic response of bacterial cells was triggered.

The modified capillary assay was performed based on the method described by Adler *et al.*^35^ to quantitatively determine the chemotaxis response of the NJN-6 strain to the banana root exudates and OA components. The NJN-6 strain was grown in LB media until an OD_600_ of 0.8 was reached. The cells collected by centrifugation were washed twice with the chemotaxis buffer described above and resuspended in the same buffer (OD_600_ = 0.8). A Petri dish 60 mm in diameter was filled with 10 mL of the cell suspension prepared above. Standard 1 μL capillaries loaded with the concentrated root exudates and three organic acids at different concentrations (10, 25, 50, and 100 μM, respectively) were immersed in the cell suspension in Petri dishes, while the chemotaxis buffer was performed as the negative control. After 30 min incubation at RT, the liquid in the capillary was transferred into a sterilized Eppendorf tube via syringe. The suspension was then diluted to 10^−3^, 10^−4^, and 10^−5^, and plated on LB plates. The CFU were determined by plating on LB plates and incubating at 37 °C for 24 h. Each treatment was replicated three times.

### Biofilm formation assay

To determine the effects of the root exudates and OAs on biofilm formation by strain NJN-6, the biofilm formation assay was performed as described by Hamon and Lazazzera^36^ in 48-well microtiter plates. The NJN-6 strain was grown in LB medium at 37 °C until an OD_600_ of 1.0 was reached. The cells were centrifuged, washed twice with 1/2 MSgg medium, and finally resuspended in 1/2 MSgg with the same volume as culture medium. Each well was filled with 1 ml 1/2 MSgg media and inoculated with 10 μL of the suspension prepared above. The negative control consisted of the culture medium alone. Either 20 μL of concentrated root exudatesor various OAs were added to the media in the wells to obtain a final concentration of 10, 25, and 50 μM. Each treatment was replicated four times. Following an incubation at 37 °C for 24 and 48 h, the biomass of the biofilms formed by NJN-6 was harvested from the 48-well plate for quantitative measurements and quantification of biofilm formation-related genes. For biomass quantitative measurements, growth medium and non-adherent cells were removed and the remaining biofilm cells were washed with distilled water. The cells were then stained with 1 mL of 0.1% crystal violet for 30 min at RT. The excess crystal violet was poured out and the wells were washed twice with distilled water; the bound crystal violet was further solubilized with 1 mL of 4:1 (v:v) ethanol and acetone acid. To quantify biofilm formation, the multi-functional plate reader Spectra Max M5 analysis system (Molecular Devices Corporation, CA, USA) was used to measure the OD_570_ of the solution in each well.

### Statistical analysis

Differences among the treatments were calculated and statistically analyzed using the analysis of variance (ANOVA) and Duncan’s multiple range test (*p* < 0.05). Statistical Package for the Social Sciences (SPSS) version 19.0 was used for statistical analysis (SPSS Inc., Chicago, IL).

### Transcription analysis of biofilm formation genes

Cells obtained from the biofilm formation experiment were induced by the application of 25 μM concentrations of each OA used for RNA isolation. RNA was isolated from *B. amyloliquefaciens* NJN-6 which formed biofilms in MSgg medium after 24 and 48 h as previously described. Total RNA samples were extracted using an RNAiso Plus kit (TaKaRa, Dalian, China) based on the manufacturer’s protocol. Then a 20 μL reverse transcription system (TaKaRa) was set up based on the manufacturer’s protocol to reverse transcribe the isolated RNA into cDNA.

Quantitative reverse transcription PCR (qRT-PCR) was used to evaluate the transcription levels of biofilm formation-related genes *yqxM* and *epsD* of strain NJN-6 by the method of Xu *et al.*^31^. We chose the *recA* gene as the internal control. All the primers involved in the experiment were reported by Xu^31^ and listed in Table S1. The reaction mixtures was consisted of a final concentration of 10 μL SYBR^@^ Premix Ex TaqTM (TaKaRa), 0.4 μL each primer (10 μM), 0.4 μL ROX Reference Dye II (50×), 0.4 μL template cDNA obtained above and 6.8 μL sterile water. Reactions were performed using the ABI 7500 system with the following conditions: 10 s at 95 °C → 95 °C for 5 s and 60 °C for 34 s of 40 cycles. To analyze the real-time PCR data the 2^−ΔΔCT^ method was used as previously reported^37^.

## Additional Information

**How to cite this article**: Yuan, J. *et al.* Organic acids from root exudates of banana help root colonization of PGPR strain *Bacillus amyloliquefaciens* NJN-6. *Sci. Rep.* **5**, 13438; doi: 10.1038/srep13438 (2015).

## Supplementary Material

Supplementary Information

## Figures and Tables

**Table 1:** Effect of various organic acids with different concentration on biofilm formation of SQR9 in 1/2 MSgg.

Treatment	Concentration	Biofilm formation of NJN-6(OD_570_, 24 h)	Biofilm formation of NJN-6(OD_570_, 48 h)
Control		1.882 ± 0.152 de	2.820 ± 0.320 c
Oxalic acid	10 μM	1.772 ± 0.139 e	2.813 ± 0.258 c
	25 μM	1.861 ± 0.301 de	2.653 ± 0.308 c
	50 μM	2.071 ± 0.347 bcde	2.951 ± 0.404 c
Malic acid	10 μM	1.974 ± 0.143 cde	2.653 ± 0.278 c
	25 μM	1.950 ± 0.373 cde	2.860 ± 0.295 c
	50 μM	1.806 ± 0.166 e	3.107 ± 0.278 bc
Fumaric acid	10 μM	2.324 ± 0.202 abcd	2.968 ± 0.342 c
	25 μM	2.589 ± 0.185 a	3.596 ± 0.420 ab
	50 μM	2.374 ± 0.374 abc	3.672 ± 0.083 a

Each treatment contained four replicates. Data were expressed as mean ± standard error. The data in a table with different letters represent significance according to the Duncan test (*p* < 0.05).
