# Supplementary material for: Organic acids from root exudates of banana help root colonization of PGPR strain Bacillus amyloliquefaciens NJN-6
Source: Sci Rep. 2015 Aug 24;5:13438. doi: 10.1038/srep13438 (PMC4547103; doi:10.1038/srep13438)
Supplement: Supplementary Information [file srep13438-s1.pdf]

Organic acids from root exudates of banana help root colonization of PGPR strain *Bacillus amyloliquefaciens* NJN-6

Jun Yuan[a1, 2], Nan Zhang[a1], Qiwei Huang[1], Waseem Raza[1], Rong Li[1], Jorge M. Vivanco[2], Qirong Shen

Table S1 Primers used for quantitative reverse transcription PCR (qRT-PCR) in this study.

Gene	Name	Sequence	Product size	Reference
recA	*recA-F*	AAAAAACAAAGTCGCTCCTCCG	109 bp	31
	recA-R	CGATATCCAGTTCAGTTCCAAG		
epsD	*epsD-F*	CAGGACAACGGCTACGACATGA	389 bp	31
	epsD-R	AGCGGGATAAACGAGAATAAAT		
yqxM	*yqxM-F*	ATTTTTACGGCTTTCGTTCATT	269 bp	31
	yqxM-R	GTCCGCTCTTTTCCCTTATTCT		
